# The Effects of Compensatory Scanning Training on Mobility in Patients with Homonymous Visual Field Defects: Further Support, Predictive Variables and Follow-Up

**DOI:** 10.1371/journal.pone.0166310

**Published:** 2016-12-09

**Authors:** Gera A. de Haan, Bart J. M. Melis-Dankers, Wiebo H. Brouwer, Oliver Tucha, Joost Heutink

**Affiliations:** 1 Department of Clinical and Developmental Neuropsychology, University of Groningen, Groningen, The Netherlands; 2 Royal Dutch Visio: Centre of Expertise for Blind and Partially Sighted People, Haren, The Netherlands; 3 Department of Neurology, University Medical Center Groningen, Groningen, The Netherlands; Universitat Regensburg, GERMANY

## Abstract

**Introduction:**

People with homonymous visual field defects (HVFD) often report difficulty detecting obstacles in the periphery on their blind side in time when moving around. Recently, a randomized controlled trial showed that the InSight-Hemianopia Compensatory Scanning Training (IH-CST) specifically improved detection of peripheral stimuli and avoiding obstacles when moving around, especially in dual task situations.

**Method:**

The within-group training effects of the previously reported IH-CST are examined in an extended patient group. Performance of patients with HVFD on a pre-assessment, post-assessment and follow-up assessment and performance of a healthy control group are compared. Furthermore, it is examined whether training effects can be predicted by demographic characteristics, variables related to the visual disorder, and neuropsychological test results.

**Results:**

Performance on both subjective and objective measures of mobility-related scanning was improved after training, while no evidence was found for improvement in visual functions (including visual fields), reading, visual search and dot counting. Self-reported improvement did not correlate with improvement in objective mobility performance. According to the participants, the positive effects were still present six to ten months after training. No demographic characteristics, variables related to the visual disorder, and neuropsychological test results were found to predict the size of training effect, although some inconclusive evidence was found for more improvement in patients with left-sided HVFD than in patients with right-sided HFVD.

**Conclusion:**

Further support was found for a positive effect of IH-CST on detection of visual stimuli during mobility-related activities specifically. Based on the reports given by patients, these effects appear to be long-term effects. However, no conclusions can be drawn on the objective long-term training effects.

## Introduction

Homonymous visual field defects (HVFD) are caused by postchiasmatic brain damage and in most cases do not fully recover [1–3]. People with HVFD often report difficulty detecting obstacles located in the blind periphery in time when moving around. This may result in feelings of insecurity and even in collisions and may have a serious impact on participation in society [4].

Several training programs have been developed aimed at optimal compensation for the HVFD by adapting eye movements. One of these, the IH-CST (InSight-Hemianopia Compensatory Scanning Training), trains patients to apply a systematic, wide horizontal scanning rhythm. Recently, a randomized controlled trial (RCT) [5] showed that the IH-CST specifically improves detection of peripheral stimuli and avoiding obstacles when moving around, especially in dual task situations. No evidence was found for an improvement in visual functions (including visual field size), reading or searching for targets on a display.

In this paper, the within-group effects of the IH-CST are analyzed in a larger patient group than in the previously reported RCT [5]. Performance of patients with HVFD at the pre-assessment and post-assessment are compared with the performance of a healthy control group. The results of a follow-up assessment are reported, as well as the associations between subjective and objective performance. Furthermore, it will be examined whether the changes between the pre-assessment and post-assessment can be predicted by certain factors. As concluded in a recent systematic review of the literature on HVFD [6], more research is needed on the predictive variables of training effects. Knowing which variables are related to the effects of training would enable rehabilitation workers to deploy the best rehabilitation program for the individual patient. This may improve efficacy of rehabilitation. The potentially predictive variables are selected for their clinical relevance. In a rehabilitation setting, it is often questioned whether training effects can be predicted by demographic characteristics, variables related to the visual disorder, and neuropsychological test results [6]. In short, the aim of the present study is to examine the effects of IH-CST in terms of subjective and objective mobility-related measures, including the long-term effects and the influence of several factors on the effect of training.

## Materials and Methods

Descriptions of the participant recruitment, training protocol and assessment measures are described in more detail elsewhere [5].

### Ethics

The Medical Research Ethics Committee of the University Medical Center Groningen (registration number METc 2010/078) and the relevant patient organizations approved the study protocol. The study was performed in accordance with the 2008 Declaration of Helsinki. Informed written consent was provided by all participants.

### Participant recruitment

Patients with a unilateral HVFD caused by acquired postchiasmatic brain injury were recruited at Royal Dutch Visio and Bartiméus, the two centers of expertise for blind and partially sighted people in the Netherlands. Patients were included if standardized ophthalmological testing confirmed the presence of a HVFD, existing for at least five months, minimum binocular visual acuity of Snellen 0.5 (6/12 or 20/40, LogMAR 0.3) and intact eye and head motility. A stable neurological and ophthalmological condition was required and patients had to be able to walk at least 50 meters independently. Furthermore, standardized neuropsychological testing was performed at Royal Dutch Visio or Bartiméus, in order to examine visual perceptual functions as well as cognitive status. Patients with severe (neuro)psychological disorders, such as neglect, or psychiatric conditions, such as anxiety disorders, were excluded from participation in the study. The MMSE score had to be ≥ 24. The results from this neuropsychological testing were collected for further analyses in case the patient was included in the study.

Healthy control participants responded to public announcements and received a financial incentive. Inclusion criteria for the healthy control group were the absence of visual, physical, neurological or psychological impairments, as confirmed by the participants during an interview. A binocular visual acuity of at least Snellen 0.8 (6/7.5 or 20/25, LogMAR 0.1) was required and their MMSE score had to be ≥ 24. Healthy control participants were selected in such a way that the distributions of age (mean, standard deviation and range) and education (proportional distribution of participants over education levels) for the control group were similar to the patient group.

### Design

Patients were assessed in the week before onset of training (T-pre) and after 13 weeks of training (T-post). More specifically, time between T-pre and T-post was filled with an introductory meeting in which the patient and occupational therapist agreed on the specific goals for training, followed by 10 weeks of training, and some spare time for delay. Training was extended with a number of sessions after T-post, i.e. outside the scope of this study, in case the individual mobility goals were not fully reached after 13 weeks of training. During a follow-up assessment (FU) six to ten months after T-post, questionnaires were administered measuring the impact of the HVFD on daily living. The scanning and mobility-related tests included in T-pre and T-post were also administered in a healthy control group. The healthy controls performed these tests however only once. Comparing performance of patients before and after training to the performance of a healthy control group puts the performance and improvement of the patients in perspective of a bench-mark.

### Relation to RCT analysis

**Fig 1:**
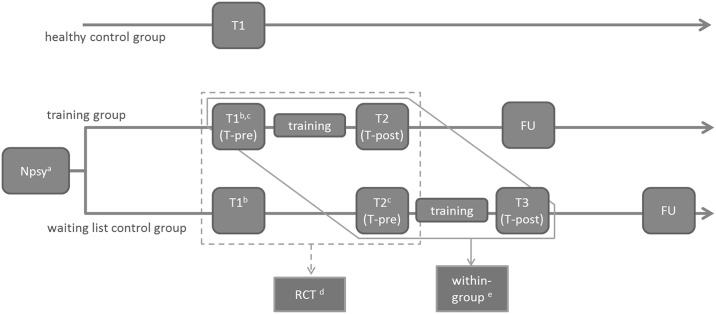
Full study design. ^a^ Neuropsychological testing in the inclusion phase including Mini Mental State Examination [7], Trailmaking Test [8], Complex Figure of Rey [9], and Hospital Anxiety and Depression Scale [10]; ^b^ including the neuropsychological tests Nederlandse Leestest voor Volwassenen [11] and 15 Word Test [12,13]; ^c^ including the neuropsychological test Digit Span (subtest of the WAIS) [14]; ^d^ treatment—non-treatment comparison (RCT) [5]; ^e^ within-group comparison of training effect (analysis described in the present paper); FU = follow up.

The participants described in this report are the same as described in the article on the RCT [5]. Fig 1 presents the full study design.

In the RCT [5], patients were assigned to either a training group or a waiting list control group. Allocation to the groups was done by the method of minimization, minimizing bias regarding important patient characteristics [5]. Both patient groups eventually received the IH-CST. Between T-pre and T-post, both patient groups followed the same training program in the same amount of time, and were handled equal in all other aspects.

From a rehabilitation perspective, it is important to know which training effects are expected and to what extent the expected training effects depend on demographic characteristics, variables related to the visual disorder, and neuropsychological test results. Also, it is important to know to what extent training effects last over time. To this end, we pooled the data from the two groups in the current analyses.

### Training

The training program is described in more detail elsewhere [5]. All patients underwent the IH-CST (InSight-Hemianopia Compensatory Scanning Training) between T-pre and T-post. The main focus of the IH-CST was on learning a systematic, anticipatory scanning rhythm that could be applied in a wide range of mobility-related activities.

The training program contained exercises aimed at improvement of awareness of the visual field defect and its consequences for daily life, learning to apply a predefined systematic scanning rhythm, and practice of the scanning rhythm in daily life mobility situations. The scanning rhythm consisted of a triad of horizontal eye movements. First, a large saccade from the center towards the blind side was made, in order to shift the visual field defect and to receive the visual information from the periphery. Then a second saccade was made back towards the seeing side to prevent overcompensation. Third, a small saccade was made back to the center. This scanning pattern was repeated at a speed matching the environmental demands and speed of moving around.

The IH-CST was developed at Royal Dutch Visio. Training was provided by occupational therapists at one location of Bartiméus and nine locations of Royal Dutch Visio in the Netherlands. There were no differences between the locations with regard to logistics, case handling, or financing of care. The IH-CST consisted of 15 sessions (18.5 hours of face-to-face training, plus homework assignments) during a period of 10 weeks by default.

### Assessments

#### Assessment procedure

The assessments were performed at the University Medical Center Groningen, the Netherlands. All participants were tested individually. Training for the patient group was provided between March 2010 and October 2012. The results of the assessments had no influence on the way the patient was treated at the rehabilitation center; data were anonymized and not provided on the individual level to Royal Dutch Visio or Bartiméus. The healthy control group started with the test for visual acuity and the MMSE and continued only if the inclusion criteria were met with regard to these tests, i.e. binocular visual acuity of at least Snellen 0.8 (6/7.5 or 20/25, LogMAR 0.1) and MMSE score ≥ 24. Participants in this group performed the tests related to scanning and mobility, with similar setup and instructions as the patient group. Assessments of the healthy control group took place between October and December 2012. All patients were approached for a follow-up six to ten months after T-post. The same questionnaires as included in T-pre and T-post were administered via telephone by research assistants who were not involved in data analyses. The follow-up assessments were performed between May 2011 and May 2013.

#### Tests for visual functions

Monocular visual acuity was measured with the ETDRS 2000 Letter Chart [15] and peak contrast sensitivity was tested with the Gecko Test [16]. Goldmann perimetry (isopters V-4, III-4 and I-4) was used to plot the monocular visual fields. Size of the intact binocular visual field was calculated and expressed in Functional Field Score, which corresponds to the number of points of a pre-defined overlay grid that fall within the intact visual field [17,18]. Furthermore, it was analyzed whether the border between the blind and intact area had shifted more than 5 degrees between T-pre and T-post. The tests for visual functions were only administered in the patient group. For the healthy control group, it was only checked if binocular visual acuity was higher than Snellen 0.8 (6/7.5 or 20/25, LogMAR 0.1).

#### Reading tests

Reading speed, minimal readable text size, and comprehension of the text were assessed with the Radner reading chart [19,20] and with a text of approximately 400 words (three standardized parallel versions).

#### Basic scanning tests

Three basic scanning tests were administered, presenting the stimuli on a large screen (40° horizontally and 33° vertically, viewing distance 192 cm). In the dot counting test, participants were asked to count dot patterns as quickly and as correct as possible. Half of the trials contained few dots (between 6 and 9 dots), while the other half contained many dots (between 18 and 21 dots). In the parallel search test, participants had to indicate whether or not the target letter O was present among T’s, again as fast and accurate as possible. In the serial search test, participants indicated whether the target letter G was present among C’s. Reaction times and accuracy scores were recorded.

#### Hazard perception test

Photos of traffic situations were presented on a large screen (40° horizontally by 25° vertically, viewing distance 192 cm). Participants were asked to carefully view the photos, each presented for eight seconds, and decide whether they would brake, release the accelerator or keep the same speed (i.e., no intervention), imagining that they were positioned in the driver’s seat. The number of incorrect responses (absolute error rate) was recorded. Two other parameters were calculated taking the type of errors into account. The adapted error rate was defined by the sum of the incorrect responses, but very risky responses (“no intervention” when the correct response is “braking”) and very cautious responses (‘braking” when the correct response is “no intervention”) were counted double. The risk-index reflected the proportion of risky answers in the adapted error rate (risk-index = (risky responses + 2*very risky responses) / adapted error rate). The hazard perception test is described in more detail by Vlakveld [21] and De Haan et al. [5]

#### Tracking Task

In the single task condition of the Tracking Task, participants had to indicate as fast and accurate as possible the pointing direction of arrows that were presented in the left or right periphery. In the dual task condition, a second task was added. While responding to the peripheral targets, participants simultaneously had to perform a steering task. On a monitor in front, a straight road was presented, on which they were driving with fixed speed. Because of an imaginary cross-wind, they had to attend to the screen continuously and correct their lateral position on the road using a steering wheel. The Tracking Task is described in more detail by Brouwer [22] and De Haan et al. [5] Outcome parameters were number of omissions (no response to stimulus), number of errors (incorrect response to stimulus) and reaction times for the peripheral stimuli, and the standard deviation in lateral position on the road (SDLP), all from the dual task condition. Dividing the mean reaction time in the dual task condition by the mean reaction time in the single task condition resulted in the dual-to-single-task-ratio (DSR).

#### Obstacle course

A standardized obstacle course was used to examine the influence of obstacles and cognitive load on walking speed. First, preferred walking speed was measured in an obstacle-free corridor. Subsequently, participants walked through the empty corridor again, but this time they had to repeat verbally presented series of digits during their walk (i.e., with cognitive load; cognitive dual task). After that, the corridor was filled with obstacles and participants walked through the course once with cognitive load and once without cognitive load. Included in the analyses were the number of contacts with obstacles and the Digit Score (proportion correct answers on the digit series) during the walk through the obstacle course. Furthermore, percentage preferred walking speed was included (PPWS = (walking speed in obstacle course with cognitive load / walking speed in empty corridor with cognitive load) * 100).

#### Questionnaires

The impact of the HVFD on activities and participation in daily life was assessed using the Independent Mobility Questionnaire (IMQ) [23], the Visual Functioning Questionnaire (NEI-VFQ-25) [24,25], and the Cerebral Visual Disorders questionnaire (CVD; described by Kerkhoff and colleagues [26] and by Dittrich, 1996, as cited by Tant [27], p.75). The three total scores of the questionnaires were included in the analyses. For the IMQ and CVD, higher scores indicate more difficulty as experienced by the patient. For the NEI-VFQ-25, higher scores mean less difficulty experienced in daily life.

### Analysis

Differences in participant characteristics between the patient group and the healthy control group were analyzed with two-tailed independent samples t-Tests for age and level of education and a two-tailed Chi-Square Test for gender.

To compare test performance of the patient group at T-pre and at T-post with test performance of the healthy control group, two-tailed independent samples t-Tests were used. Change within the patient group between T-pre and T-post was examined with two-tailed matched pairs t-Tests. For the questionnaire data in the patient group, the results of T-pre, T-post, and FU were compared in a General Linear Model (GLM) Repeated Measures analysis, with simple contrasts comparing T-pre with FU and T-post with FU. In case Levene’s test showed that the assumption of equal variances could not be assumed for a t-Test, the unequal-variance t-Test was used. Missing values were excluded pairwise. Significant effects were defined by *P*-values < .05. *P*-values are reported in case of a *P*-value < 0.10. Cohen’s d was used for calculating the effect sizes of the between-group and within-group comparisons [28]. Effect sizes were classified as negligible (d < 0.20), small (0.20 < d <0.50), medium (0.50 < d < 0.80) or large (d > 0.80).

Four parameters related to scanning in mobility situations were further examined. These outcome parameters were found to improve by IH-CST in the RCT analysis [5]. The total score on the IMQ reflected the experienced difficulty in several mobility situations as reported by the patients, reaction time to peripheral stimuli in the dual tracking task (TT-RT-all) was an indication of the level of compensation regarding detection of information in the periphery, the DSR in the tracking task (DSR-all) represented the influence of a secondary task on efficiency of scanning, and PPWS represented a measure of the ability to detect and avoid obstacles during walking. First, Pearson’s correlations were used to examine how self-reported mobility performance was related to test performance. Then, a linear regression analysis was performed for each of the outcome parameters to examine the associations between training effects and a number of demographic characteristics, variables related to the visual disorder, and neuropsychological test results. The absolute difference between scores on T-pre and T-post was chosen as the dependent variable reflecting training effect. The independent variables, including score of the outcome parameter on T-pre, were inserted stepwise. Listwise exclusion in case of missing values would have led to few remaining data. Therefore, multiple imputation [29,30] was applied for the missing values among the independent variables. This created multiple datasets (five in this case) with different values for the originally missing values. Each regression analysis was then performed on each of these five datasets, resulting in one final model in which the five outcomes were pooled back together.

## Results

The individual-level data are provided in S1 File.

### Participants

Nine of the 54 included patients with HVFD dropped out of the study after T-pre. One patient had deceased and other reasons for drop-out were health problems (n = 2), difficulties scheduling the training or assessments (n = 2), or too low compliance with the training protocol (n = 4). For 35 of the remaining patients, brain infarction was the cause of the HVFD. Other causes of the HVFD were hemorrhagic vascular accident (n = 3), traumatic brain injury (n = 2), penetrating head trauma (n = 1), extirpation of arteriovenous malformation with postoperative hemorrhage (n = 1), or combined etiology (n = 3). For 19 patients, T-pre was the second time they performed the tests. These patients had participated in the early pre-assessment 13 weeks prior to start of the training as part of the RCT [5] (see Fig 1). Table 1 summarizes the participants’ characteristics (level of education according to Verhage [31]; higher values represent higher levels of education). There were relatively fewer men in the healthy control group compared to the patient group (χ^2^(1) = 9.64, *P* = 0.003).

**Table 1:** Summary of participant characteristics (numbers, mean ± SD, range).

	Patient group (n = 45)	Healthy group (n = 25)	*P*-value
Gender	30 men, 15 women	7 men, 18 women	.003 ^a^ (Chi^2^ Test)
Age	55 ± 10.9 [27;74]	53 ± 14.5 [28;76]	.530 (t-Test)
Level of education	5.4 ± 0.8 [4;7]	5.5 ± 0.8 [4;7]	.494 (t-Test)
Side of HVFD	31 left HVFD, 14 right HVFD		
Functional Field Score	60 ± 9.1 [44;80], 10 quadrantanopia, 35 hemianopia		
Time since onset of HVFD (months)	22 ± 24.4 [5;122]		

^a^ Significant difference between the patient group and the healthy control group (*P* < .05).

Royal Dutch Visio provided training for forty-four patients and Bartiméus for one patient. Training was extended after T-post for 10 patients. Time between neuropsychological testing and T-pre was on average 19 weeks (range 2–68 weeks). For one patient, time between T-pre and T-post was 16 instead of 13 weeks, because of difficulties rescheduling T-post). Follow-up data could not be collected for two patients; one patient was not willing to participate and one patient had deceased between T-post and FU. The average time lag between T-post and FU was 8.5 months (range 6.5–14.6). For two patients, time between T-post and FU exceeded 10 months (10.6 and 14.6 months respectively).

### Training effects

Mean test scores and standard deviations, as well as effect sizes corresponding to the different t-Tests are presented in Table 2.

**Table 2:** Mean test scores (SD) and effect sizes for group differences (absolute values).

	Test scores						Effect sizes		
	Healthy control group		Patient group before training (T-pre)		Patient group after training (T-post)		patient group T-pre vs. healthy control group	patient group T-pre vs. T-post	patient group T-post vs. healthy control group
	n	mean (SD)	n	mean (SD)	n	mean (SD)			
**Tests for visual functions**									
Visual acuity right eye	-		45	0.94 (0.27)	45	0.93 (0.26)	-	0.04	-
Visual acuity left eye	-		45	0.97 (0.25)	45	0.96 (0.24)	-	0.05	-
Peak contrast sensitivity	-		45	1.93 (0.16)	45	1.94 (0.15)	-	0.08	-
Functional Field Score	-		45	59.83 (9.14)	45	59.40 (9.01)	-	0.08	-
**Reading tests**									
Radner average reading speed (wpm)	-		43	151.4 (32.56)	43	156.2 (35.94)	-	0.23	-
Minimal readable text size (LogRad)	-		43	0.07 (0.14)	43	0.08 (0.11)	-	0.17	-
Text reading speed (wpm)	-		42	134.7 (28.58)	42	135.6 (28.59)	-	0.05	-
Text correct answers	-		43	1.51 (0.59)	43	1.67 (0.47)	-	0.22	-
**Basic scanning tests**									
*Dot counting test*									
Reaction times (ms)									
All trials	25	6631 (1496)	40	**8634 (3255)** a	40	**8132 (3242)** a	**0.74**	0.17	**0.55**
Few dots	25	3214 (818)	40	**4832 (1870)** a	40	**4576 (1837)** a	**1.04**	0.16	**0.89**
Many dots	25	10048 (2347)	40	**12427 (5071)** a	40	11700 (5120)	**0.56**	0.16	0.39
Proportion correct answers									
All trials	25	0.84 (0.12)	40	**0.74 (0.20)** a	40	**0.75 (0.18)** a	**0.60**	0.13	**0.54**
Few dots	25	0.96 (0.07)	40	0.91 (0.18)	40	0.92 (0.17)	0.34	0.04	0.31
Many dots	25	0.72 (0.21)	40	**0.56 (0.28)** a	40	0.59 (0.27)	**0.62**	0.14	**0.51**
*Parallel search test*									
Reaction times (ms)									
All trials	25	1196 (367)	40	**2264 (687)** a	40	**2142 (720)** a	**1.82**	0.17	**1.55**
Target present	25	996 (260)	40	**1477 (481)** a	40	**1382 (397)** a	**1.17**	0.22	**1.10**
Target absent	25	1396 (504)	40	**3049 (936)** a	40	**2904 (1091)** a	**2.07**	0.13	**1.65**
Accuracy									
Total number of errors	25	0.48 (0.77)	39	0.64 (1.33)	39	0.49 (0.79)	0.14	0.14	0.01
Number of omissions	25	0.32 (0.63)	39	0.44 (1.23)	39	0.36 (0.74)	0.12	0.09	0.06
*Serial search test*									
Reaction times (ms)									
All trials	25	3498 (1337)	40	**5443 (1982)** a	40	**5177 (1733)** a	**1.10**	0.18	**1.05**
Target present	25	2600 (1321)	40	**3750 (1588)** a	40	**3634 (1309)** a	**0.77**	0.11	**0.79**
Target absent	25	4395 (1474)	40	**7136 (2625)** a	40	**6721 (2343)** a	**1.21**	0.20	**1.13**
Accuracy									
Total number of errors	25	0.84 (1.38)	40	1.23 (1.63)	40	1.70 (1.91)	0.25	0.22	**0.50**
Number of omissions	25	0.76 (1.39)	40	1.10 (1.41)	40	1.48 (1.77)	0.24	0.21	0.44
**Hazard perception test**									
Absolute error rate	24	8.50 (2.21)	36	9.36 (2.79)	36	8.83 (2.54)	0.33	0.19	0.14
Adapted error rate	24	9.21 (2.89)	36	10.50 (3.48)	36	9.64 (2.94)	0.40	0.28	0.15
Risk-index	24	0.76 (0.18)	36	0.72 (0.17)	36	0.75 (0.16)	0.19	0.14	0.07
**Tracking Task**									
*Dual task condition*									
Reaction times (ms)									
All stimuli (TT-RT-all)	25	943 (147)	41	**1219 (266)** a	41	**1135 (231)** a**,**^b^	**1.21**	0.36	**0.94**
Stimuli blind side	-		41	1530 (505)	41	**1332 (338)** b	-	0.48	-
Stimuli seeing side	-		41	1047 (204)	41	1015 (237)	-	0.18	-
Stimuli blind side—stimuli seeing side	-		41	483 (399)	41	**318 (309)** b	-	0.44	-
Accuracy									
Number of faulty responses	25	0.84 (1.07)	42	0.71 (0.97)	42	0.86 (0.93)	0.13	0.13	0.02
Number of omissions	25	0.04 (0.20)	42	0.45 (1.31)	42	**0.10 (0.37)** b	0.39	0.32	0.19
Standard Deviation of Lateral Position (SDLP)	25	46.1 (6.50)	40	48.2 (10.72)	40	49.4 (10.49)	0.22	0.14	0.35
*Mean reaction time dual task divided by mean reaction time single task (dual-to-single-task-ratio*, *DSR)*									
All stimuli (DSR-all)	25	1.00 (0.11)	40	**1.25 (0.22)** a	40	**1.14 (0.21)** a**,**^b^	**1.35**	0.48	**0.79**
Stimuli blind side	-		40	1.56 (0.53)	40	**1.30 (0.41)** b	-	0.42	-
Stimuli seeing side	-		40	1.07 (0.19)	40	1.07 (0.19)	-	0.02	-
**Obstacle course**									
Digit Score	25	0.79 (0.24)	43	**0.64 (0.30)** a	43	0.68 (0.30)	**0.56**	0.21	0.42
Number of contacts	25	0.48 (0.65)	43	**1.74 (1.79)** a	43	**0.74 (1.03)** b	**0.85**	**0.68**	0.29
PPWS	25	58.0 (7.88)	43	**48.1 (10.32)** a	43	**50.6 (9.39)** a**,**^b^	**1.04**	0.34	**0.84**
**Questionnaires**									
NEI-VFQ-25 total score	-		45	64.38 (14.00)	45	**71.03 (11.40)** b	-	**0.59**	-
IMQ total score	-		45	2.52 (0.71)	45	**2.08 (0.51)** b	-	**0.75**	-
CVD total score	-		45	0.46 (0.16)	45	**0.36 (0.13)** b	-	**0.58**	-

^(a)^ significant difference between patient group and healthy control group (independent sample t-Test. two-sided *P*-value < .05).

^(b)^ significant difference between T-pre and T-post within patient group (matched pairs t-Test. two-sided *P*-value < .05).

#### Tests for visual functions

For the visual functions tests, no significant differences were found between T-pre and T-post (all *P* > .100, all negligible effect sizes).

#### Reading tests

No significant differences were found on the reading tests between T-pre and T-post (all *P* > .100, all negligible effect sizes, except for small increases in Radner mean reading speed and correct answers of the standardized text). When analyses were performed separately for patients with left and right HVFD, similar results were obtained (all *P* > 0.100).

#### Basic scanning tests

At T-pre, all reaction time parameters of the basic scanning tasks were significantly higher for the patients than for the healthy controls. For most of these parameters, the difference was still present at T-post (all *P* > .014, all medium and large effect sizes). Only the counting of many dots was no longer significantly slower in patients than in healthy controls at T-post (t(58.8) = 1.77, *P* = .083, small effect size). No significant differences were found for the reaction times between T-pre and T-post in the patient group (all *P* > .100, all small or negligible effect sizes).

With regard to the accuracy rates on the dot counting test, patients made more errors than healthy controls at T-pre regarding the total number of trials (t(63) = -2.35, *P* = .022, medium effect size) and the trials with many dots specifically (t(63) = -2.43, *P* = .018, medium effect size). At T-post, this difference was still present for the total number of trials (t(63) = -2.12, *P* = .038, medium effect size), while the difference for the trials with many dots was still of medium size, but just missed significance (t(63) = -2.00, *P* = .050). For the trials with few dots, no differences were found between the two groups for T-pre or T-post (both *P* > .100 and small effect sizes). No changes were found between T-pre and T-post for the accuracy scores on the dot counting task (all *P* > .100 and of negligible size).

For the accuracy rates of the visual search tests, no between-group or within-group effects were found (all *P* > .100, all small or negligible effect sizes, except for a medium sized difference between patients and healthy controls at T-post for the total number of errors on the serial search test).

#### Hazard perception test

No significant between-group or within-group effects were found for the accuracy rates of the hazard perception test (all *P* > .100, all small or negligible effect sizes).

#### Tracking Task

Data from the tracking task showed that after training, the number of omissions of peripheral stimuli had decreased significantly (t(41) = 2.06, *P* = .046, small effect size). Patients made more omissions than healthy controls, but this difference was of small size and not significant at T-pre (t(44.2) = -2.00, *P* = .052) and of negligible size and not significant at T-post (*P* > .100). No significant between-group or within-group effects were found for the number of incorrect responses (negligible effect sizes) and SDLP (small between-group differences and a negligible within-group difference; all *P* > .100).

**Fig 2:**
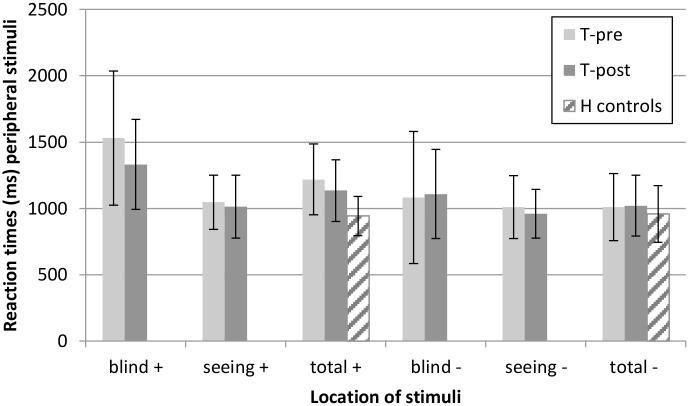
Reaction times (mean ± SD) for peripheral stimuli in the Tracking Task. Split for stimuli on the blind side and seeing side for the patient group. (+) dual task condition, (-) single task condition.

**Fig 3:**
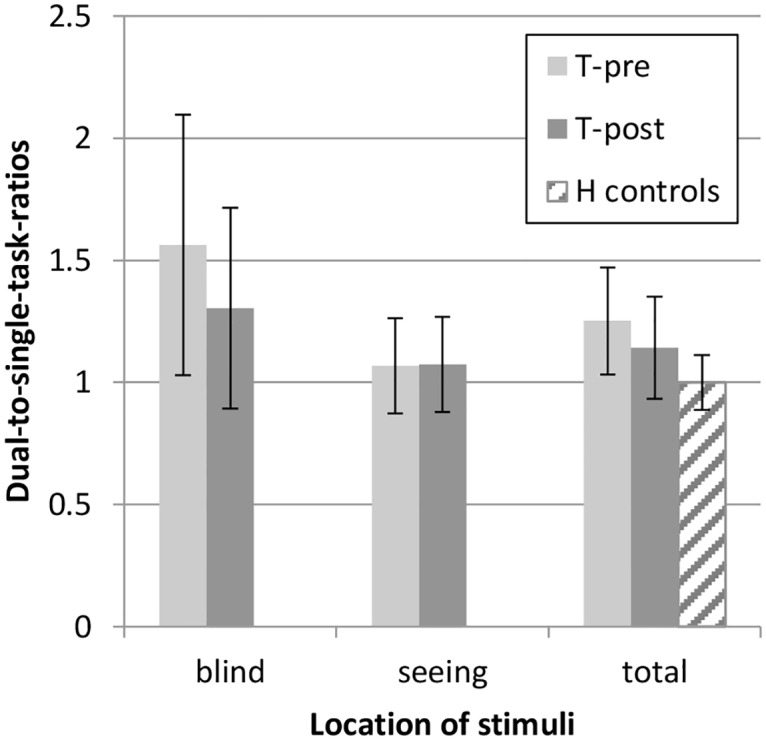
Dual-to-single-task-ratios (mean ± SD) for peripheral stimuli in the Tracking Task. Split for stimuli on the blind side and seeing side for the patient group.

Reaction times for the peripheral stimuli are presented in Figs 2 and 3. Detection of peripheral targets became faster between T-pre and T-post in the patient group (t(40) = 2.28, *P* = .028, small effect size), although the patients responded significantly slower than healthy controls at both T-pre (t(63.6) = -5.42, *P* < .001, large effect size) and T-post (t(64) = -3.71, *P* < .001, large effect size). A decrease in reaction time between T-pre and T-post was found especially for targets on the blind side (t(40) = 3.06, *P* = .004, small effect size) and not for targets on the seeing side (*P* > .100, negligible effect size). The difference in reaction times for stimuli on the blind and seeing side decreased after training (t(40) = 2.85, *P* = .007, small effect size). The dual-to-single-task-ratio (DSR) also decreased after training (t(39) = 3.02, *P* = .004, small effect size) and was significantly higher in the patient group than in the healthy control group at T-pre (t(61.1) = -6.09, *P* < .001, large effect size) as well as T-post (t(61.9) = -3.54, *P* = .001, medium effect size). DSR decreased specifically for stimuli on the blind side (t(39) = 2.67, *P* = .011, small effect size). DSR for stimuli on the seeing side was already close to 1.00 at T-pre and did not change by training (*P* > .100, negligible effect size).

#### Obstacle course

With regard to the obstacle course, the PPWS increased between T-pre and T-post (t(42) = -2.21, *P* = .033, small effect size). PPWS was significantly lower for patients than for healthy controls at both T-pre (t(66) = -4.12, *P* < .001, large effect size) and T-post (t(66) = -3.32, *P* = .001, medium effect size). The number of contacts was significantly higher for patients than for healthy controls at T-pre (t(58.1) = 4.18, *P* < .001, large effect size), decreased significantly between T-pre and T-post (t(42) = 4.43, *P* < .001, medium effect size) and was no longer significantly different between patients and healthy controls at T-post (*P* > .100, small effect size). Digit Score was significantly lower for patients than for healthy controls at T-pre (t(66) = -2.21, *P* = .031, medium effect size), but no longer at T-post (*P* > .100, small effect size), although change between T-pre and T-post was not significant (*P* > .100, small effect size).

#### Questionnaires

All three questionnaires indicated significant improvement between T-pre and T-post (all *P* < .001 and medium effect sizes).

### Follow up

**Fig 4:**
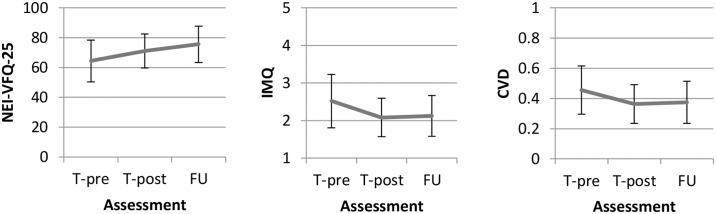
Questionnaire scores (mean and standard deviation presented) of the patient group on T-pre, T-post, and FU. Vertical axes indicate the mean total scores on the NEI-VFQ-25, the IMQ and the CVD.

The questionnaire data are presented in Table 3 and Fig 4. For all three questionnaires, test scores changed significantly between T-pre, T-post, and FU (F(2,41) = 20.25, *P* < .001). Compared to T-pre, scores on all three questionnaires had significantly improved at the time of FU (all *P* < .001, medium effect sizes for IMQ and CVD, large effect size for VFQ). Between T-post and FU, the IMQ score and CVD score did not change significantly (both *P* > .100, negligible effect sizes), while the VFQ scores indicated further improvement (F(1,42) = 12.27, *P* = .001, medium effect size). Exclusion of the two patients with 11 and 15 months (instead of 6 to 10 months) between T-post and FU led to similar results (data not presented).

**Table 3:** Mean test scores (SD) and effect sizes for group differences (absolute values) for patients that completed follow-up (n = 43).

Questionnaires	T-pre	T-post	FU	Effect size T-pre vs. FU	Effect size T-post vs. FU
NEI-VFQ-25 total score	63.62 (13.78)	70.83 (11.30)	75.55 (12.25) a,b,c	0.98	0.53
IMQ total score	2.56 (0.70)	2.08 (0.52)	2.12 (0.54) a,b	0.68	0.08
CVD total score	0.47 (0.16)	0.36 (0.13)	0.37 (0.14) a,b	0.65	0.09

^(a)^ significant overall effect of test assessment (GLM Repeated Measures, *P*-value < .001).

^(b)^ significant difference between T-pre and FU (simple contrast, *P*-value < .001)

^(c)^ significant difference between T-post and FU (simple contrast, *P*-value = .001)

### Self-reported performance related to test results

No significant correlations were found at T-pre between mobility performance in daily life as reported by the patients and mobility performance as measured in the Tracking Task and obstacle course (r_IMQ(pre)—TT-RT-all(pre)_ = .158, *P* = .307; r_IMQ(pre)–DSR-all(pre)_ = .054, *P* = .732; r_IMQ(pre)–PPWS(pre)_ = -.013, *P* = .936). Self-reported improvement in daily life mobility between T-pre and T-post was not found to be related to change in mobility performance on the Tracking Task or obstacle course (r_IMQ(diff)—TT-RT-all(diff)_ = -.078, *P* = .628; r_IMQ(diff)–DSR-all(diff)_ = .067, *P* = .681; r_IMQ(diff)–PPWS(diff)_ = .018, *P* = .910).

### Predictors of training effects

The regression output in Table 4 shows the variables that were found to relate to the size of the training effects regarding the outcome measures IMQ, TT-RT-all, DSR-all and PPWS. Inserted as independent variables were: score at T-pre, age, gender, side of HVFD, type of HVFD (hemianopia or quadrantanopia), Functional Field Score, time since onset, Mini Mental State Examination (total score) [7], Trailmaking Test (TMT-A time, TMT-B time, B/A-index) [8], Complex Figure of Rey (score on copy task) [9], Hospital Anxiety and Depression Scale (total score) [10], Nederlandse Leestest voor Volwassenen (‘ruwe score’) [11], 15 Word Test (total correct responses immediate recall, correct responses delayed recall) [12,13], Digit Span (maximum repeated numbers forward, maximum repeated numbers backward) [14].

**Table 4:** Results of regression analyses on potential predictors of training effects.

Training effect (T-pre—T-post)	Significant predictors	b	*P*-value	Regression formula
IMQ total score	IMQ_pre_	0.577	< .001	IMQ_diff_ = -1.017 + 0.577 * IMQ_pre_
TT-RT-all	TT-RT-all_pre_	0.547	< .001	TT-RT-all_diff_ = -540.199 + 0.547 * TT-RT-all_pre_− 131.880 * SideHH
	SideHH	-131.880	.037	
DSR-all	DSR-all_pre_	0.597	< .001	DSR-all_diff_ = -0.637 + 0.597 * DSR-all_pre_
PPWS	PPWS_pre_	0.331	.001	PPWS_diff_ = -18.336 +0.331*PPWS_pre_

**Fig 5:**
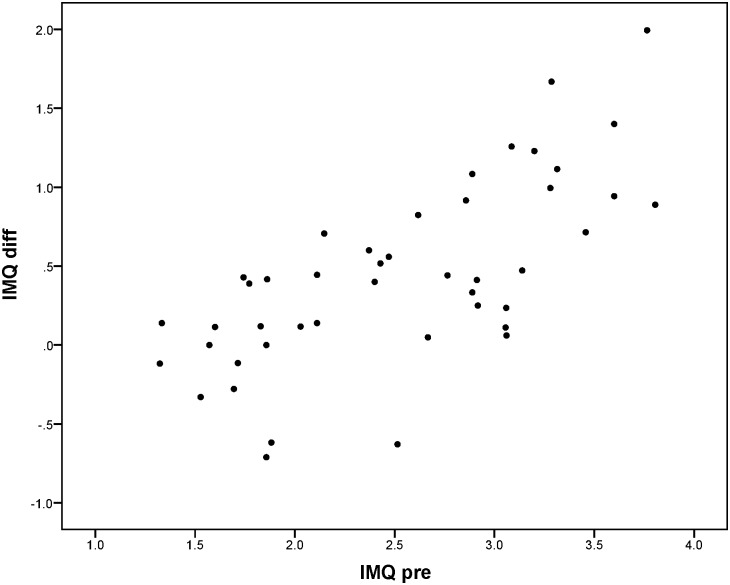
IMQ total score at T-pre (IMQ pre) vs. difference score (IMQ diff = T-pre—T-post: positive value means improvement).

**Fig 6:**
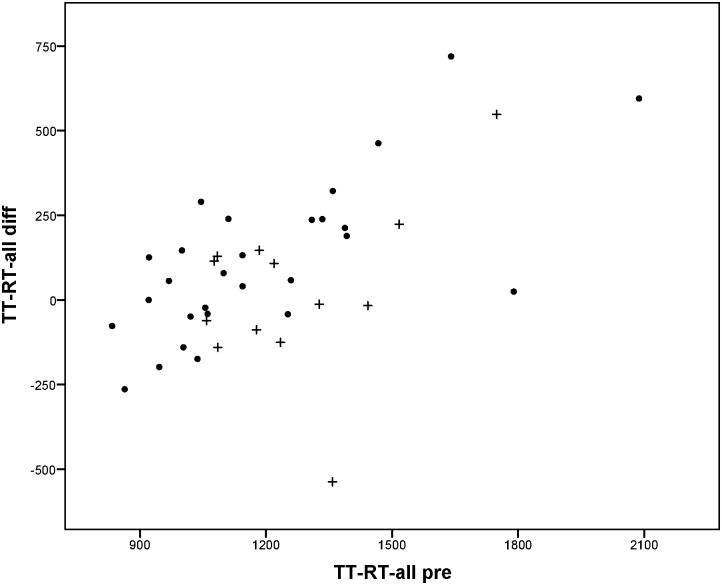
Reaction times to peripheral stimuli in the dual Tracking Task: score at T-pre (TT-RT-all pre) vs. difference score (TT-RT-all diff = T-pre—T-post: positive value means improvement). • left-sided HVFD + right-sided HVFD.

**Fig 7:**
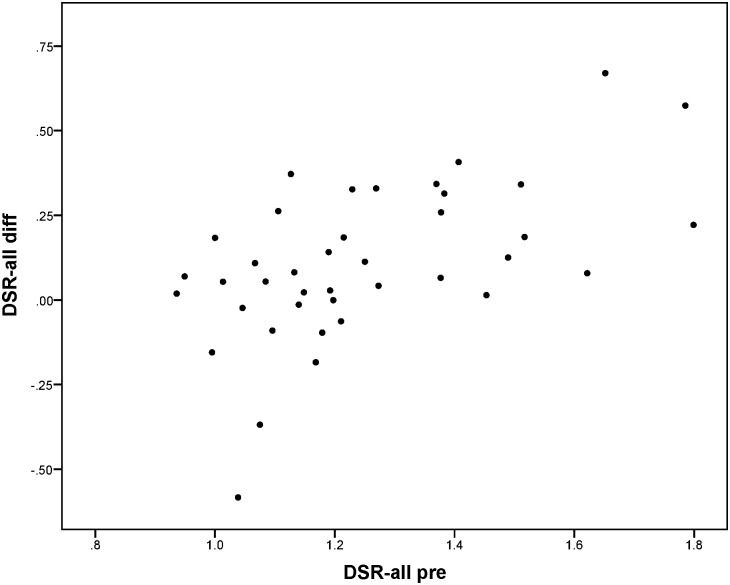
Dual-to-single-task-ratio in reaction times to peripheral stimuli in the dual Tracking Task: score at T-pre (DSR-all pre) vs. difference score (DSR-all diff = T-pre—T-post: positive value means improvement).

**Fig 8:**
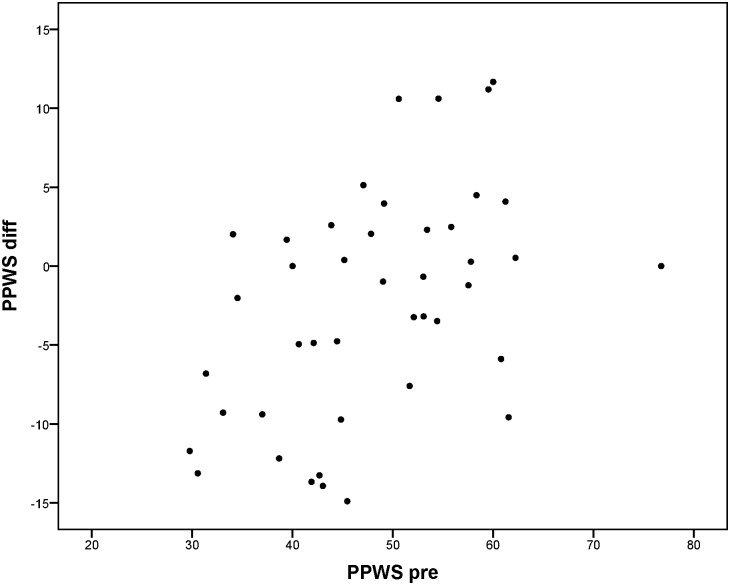
Percentage preferred walking speed in obstacle course cognitive dual task: score at T-pre (PPWS pre) vs. difference score (PPWS diff = T-pre—T-post: negative value means improvement).

Change between T-pre and T-post was mainly predicted by the scores on T-pre; patients performing worse on T-pre on average improved more than patients performing better on T-pre. For the reaction times to peripheral stimuli in de Tracking Task (TT-RT-all), it was found that besides the score on T-pre, side of the HVFD was also related to the extent of improvement. Reaction times decreased more for patients with left-sided HVFD than for patients with right-sided HVFD. No other variables were found to be significant predictors in the regression models. Although the plots in Figs 5 to 8 show that for every outcome parameter there were a few people who performed worse at T-post than at T-pre, there were no T-pre values for which all participants showed a decline in performance.

## Discussion

This study evaluated the effects of compensatory scanning training (IH-CST) for patients with HVFD. First, it was examined whether the results of an RCT on the effect of IH-CST [5] could be confirmed in a larger patient sample by comparing data from T-pre and T-post and by comparing the patient group with a healthy control group. Second, it was evaluated whether there is evidence for long-term training effects. The third aim of this study was to determine whether training effects could be predicted by demographic characteristics, variables related to the patients’ visual disorder, and neuropsychological test results. This would allow clinicians working in the field of rehabilitation to select appropriate and effective rehabilitation programs for their patients.

The results of the RCT [5] showed that compared to a waiting list control group, patients who received the IH-CST specifically improved in detecting peripheral stimuli in mobility situations, while no effects were found on visual functions (including visual field size), reading, visual search or dot counting. Analyzing the data from T-pre and T-post in an extended patient group (which was created by merging the data of the two groups of the RCT analysis [5] as explained in the method section) provided further support for these conclusions. The within-group training effects in the current study were very similar to the within-group training effects in the training group of the previously reported RCT. Only a few differences between the results of the two analyses were found, which are described in Table 5.

**Table 5:** Differences between the results of the current analyses and the previous RCT analyses [5].

Parameter	Description of differences between the results
Tracking Task: number of omissions of peripheral stimuli, reaction times for stimuli on the blind side, and difference in reaction times between the blind and seeing side	The analysis of the current data revealed significant improvements that were not found to be significant in the smaller sample of the RCT.
Standardized reading text: number of correct answers	The finding of the RCT that the number of correct answers on the standardized reading test increased significantly after training was not confirmed by the current analysis.
Tracking Task: dual-to-single-task-ratio (DSR-all)	Regarding differences of effect sizes of the within-group training effects (in terms of effects of smaller or larger than 0.50), only one difference was found between the RCT analysis and the current analysis. A medium effect of 0.70 was found for the improvement in dual-to-single-task-ratio (DSR-all) in the RCT, while this effect just missed the threshold value for a medium effect in the current analysis (effect size 0.48).

Although the IH-CST improved visual performance in mobility-related tests, the patient group still performed poorer on most parameters after following the training in comparison to the healthy control group. Performance in the obstacle course after training, however, was no longer significantly different from the performance of the healthy control group after training regarding the number of contacts with obstacles and the score acquired in the cognitive task during walking.

The self-reported improvements caused by training appear to be long-term effects. Based on the questionnaire data from the half year follow-up, the positive effects of training on activities and participation in daily life were still present or increased even further six months after training. However, the data from T-pre and T-post indicated that self-reported improvement did not correlate with improvement in objective mobility performance. Therefore, no conclusions can be drawn on the long-term training effects on objective measures. Since the focus of the IH-CST is on automatizing the compensatory scanning strategy as much as possible, this skill might slowly deteriorate without the patient noticing. One may consider implementing refresher sessions, e.g. six months following the training sessions and each following year.

In the present study, only patients without severe (neuro)psychological disorders, such as neglect, or psychiatric conditions, such as anxiety disorders, were included. In this group with minimal comorbidity, training effects could not be predicted by demographic characteristics, variables related to the visual disorder, or neuropsychological test results. The only variable with potential predictive value was *side of HVFD*, and by this *side of lesion*; reaction times to peripheral stimuli in a dual task situation decreased on average more for patients with a left-sided HVFD compared to patients with a right-sided HVFD. However, no influence of side of HVFD on training effect was found for the other variables. Therefore, the results are inconclusive on the effect of side of HVFD.

A recurring finding for both the subjective and the objective measures was that change between T-pre and T-post depended on performance at T-pre. Patients who performed worse before onset of training, showed in general more improvement after training in terms of the absolute difference in performance. This might be explained by a ceiling effect for those patients that already performed well prior to training. However, even the patient group with the best performance before onset of training still improved on one or more parameters. It cannot be ruled out that the larger effect of training for lower performers has its limits. For patients with extremely low performance because of serious comorbidity, such as a severe cognitive or physical disorder, this comorbidity may interfere with training effects. For example, if a person is not able to walk more than 50 meters because of physical health problems, mobility performance may not improve substantially after following the IH-CST.

Two comments are made with respect to the methods used in the present study. First, in the analyses of potential predictors of training effects, the amount of change by training was defined by the absolute difference between the scores on T-pre and T-post. We recognize that a direct translation to clinical implications is not possible and that use of other definitions of training effects may lead to different nuances in the results. Second, we did not investigate possible carry-over effects of treatment and advice in the subacute stage on the effects of the IH-CST. We cannot exclude the possibility that patients who received good explanation about the nature and consequences of the HVFD in the subacute stage profited more from training than people who received less explanations and instructions. All patients in this study had in common that despite previous explanation, advices, or treatment, they all still experienced mobility-related difficulties in daily life and signed up for help on this matter at Visio or Bartiméus. The current study examined whether the IH-CST improves mobility-related tasks and activities, in addition to previous treatment that may have been received. Future studies may investigate whether and how treatment in the subacute stage interacts with the effects of IH-CST.

With regard to clinical practice, a number of recommendations are provided. Based on the findings of the current study in combination with the results of the previous RCT [5], the IH-CST is recommended for improving detection of visual stimuli during mobility-related activities for patients with HVFD and minimal comorbidity, as assessed via neuropsychological testing. An observation of visual performance during mobility-related activities prior to training seems crucial to inform the clinician and patient about the training effects to be expected. In order to evaluate the progress made by an individual patient, it is important to assess both the training effects as experienced by the patient, as well as the changes on objective test measures. The training protocol provides standardized exercises and scoring forms for the occupational therapists to measure progress, as well as an evaluation form that is used to assess the patients’ own idea of improvement. In order to measure the objective improvement on mobility-related activities different from the exercises included in the training program, tests may be implemented in the rehabilitation center before and after training, such as a test similar to the Tracking Task of the current study. Standardized questionnaires, such as the IMQ, might be incorporated to quantify the changes as experienced by the patients.

Future studies may examine the effect of the IH-CST, or an adapted version, for patients with neglect (with or without HVFD). A recent Cochrane review [32] concluded that at the moment, no rehabilitation approach for neglect can be supported or refuted from current randomized controlled trials.

In conclusion, additional evidence was provided confirming the beneficial effect of IH-CST on detection of visual stimuli during mobility-related activities. Again, no evidence was found for improvement on visual functions, reading, visual search and dot counting. According to the patients’ reports, the effects were still present six to ten months after training. However, the patients’ impressions could not be supported by objective data since no objective measurements were included in the follow-up assessment. Besides inconclusive evidence for more improvement in patients with a left-sided HVFD than in patients with right-sided HFVD, no demographic characteristics, variables related to the patients’ visual disorders, and neuropsychological test results have been found to predict size of training effect in this sample where serious (neuro)psychological disorders have been excluded. Further research is needed to explore the effects and feasibility of IH-CST for patients with comorbidity. Also, it is recommended to examine the need for a follow-up training to ensure permanent use of the skills practiced and acquired during the IH-CST.

## Supporting Information

S1 File Individual-level data. (XLSX)
